# Occipital Pial AVM Rupture in a Young Adult: Dual Intranidal Aneurysms, Solitary Parasagittal SSS Drainage, and Hematoma-Corridor Microsurgical Cure

**DOI:** 10.3390/diagnostics16020265

**Published:** 2026-01-14

**Authors:** Alexandru Breazu, Stefan Oprea, Nicolaie Dobrin, Ionut Bogdan Diaconescu, Octavian Munteanu, Matei Șerban, Răzvan-Adrian Covache-Busuioc, Corneliu Toader, Mugurel Petrinel Rădoi, Cosmin Pantu

**Affiliations:** 1 Faculty of General Medicine, Carol Davila University of Medicine and Pharmacy, 050474 Bucharest, Romania; 2 Department of Anatomy, Carol Davila University of Medicine and Pharmacy, 050474 Bucharest, Romania; 3 Puls Med Association, 051885 Bucharest, Romania; 4 Nicolae Oblu Clinical Hospital, 700309 Iasi, Romania; 5 University Hospital, Carol Davila University of Medicine and Pharmacy, 010024 Bucharest, Romania; 6 Department of Neurosurgery, Carol Davila University of Medicine and Pharmacy, 050474 Bucharest, Romania; 7 Department of Vascular Neurosurgery, National Institute of Neurology and Neurovascular Diseases, 077160 Bucharest, Romania

**Keywords:** brain arteriovenous malformation, posterior convexity AVM, occipital pial AVM, intranidal aneurysm, intracerebral hemorrhage, hemorrhagic presentation, superficial venous drainage, superior sagittal sinus, catheter angiography, hematoma corridor

## Abstract

**Background and Clinical Significance:** Focal hemorrhagic severity associated with posterior convexity pial brain arteriovenous malformation (AVM) cases can be exacerbated by hemodynamic stress focusing on focal areas of architectural weakness and by superficial venous outflow being restricted by non-redundant superficial venous drainage. This clinical case report exemplifies how bedside neurologic localization and angioarchitectural characteristics can inform the selection of microsurgical approaches for the treatment of ruptured AVMs that are directed at reducing hemorrhage recurrence risk through corridors based on rupture location. **Case Presentation:** An otherwise healthy young adult male (modified Rankin scale [mRS] pre-morbid = 0) initially presented with a thunderclap headache, emesis, photophobia, decreased level of consciousness (admitted Glasgow Coma Score [GCS] = 11; E3V3M5), and subsequent deficits including left-sided pyramidal weakness, visual field loss, and visuo-spatial neglect. A non-contrast computed tomogram (CT) confirmed an intraparenchymal hemorrhage (ICH) located within the right hemisphere’s posterior lobe. Angiographic evaluation of this AVM with catheter injection and three-dimensional reconstruction revealed a compact right occipital posterior convexity pial AVM (nidus 8 × 3 mm) supplied by distal cortical branches of the right middle cerebral artery (MCA); all blood draining from the nidus was directed to a single cortical vein which then drained into the superior sagittal sinus; there were two additional intranidal saccular aneurysms (approximately 3 × 2 mm and 3 × 3 mm). Because of the acute worsening secondary to ICH and because all venous drainage was superficial-only, a single-stage approach was selected given the urgency: decompressive evacuation of the hematoma via a corridor to the site of the AVM, followed by microsurgical removal of the AVM. The removal of the AVM was accomplished in a feeder-first, vein-last sequence, and en-passage arteries and parasagittal bridging veins were preserved throughout the procedure. Additionally, the two intranidal aneurysms identified as potential weak points during progressive devascularization of the AVM were specifically treated during the removal procedure. Following the successful removal of the AVM, the patient experienced a rapid recovery and returned to a nearly premorbid state of functioning, excepting a persistent small area of quadrantanopia. **Conclusions**: Rupture of posterior convexity AVMs may result in increased hemorrhagic severity due to localized architectural weaknesses in addition to the overall size of the AVM nidus. By correlating neurological findings, the topography of the hemorrhage, and angioarchitectural features early after rupture, emergency decisions regarding management can be better informed. The application of a hematoma-corridor, feeder-first/vein-last microsurgical approach for the treatment of such AVMs can achieve definitive curative results while minimizing damage to posterior cortical regions.

## 1. Introduction

Arteriovenous Malformations (AVMs), pial brain arteriovenous malformations, are congenital vascular lesions characterized by direct arteriovenous communication through an abnormal vascular network (nidus) which lacks a capillary bed between the artery and vein, therefore representing an arteriovenous shunt [1]. The hemodynamics of AVMs are variable: AVMs can be classified as high flow shunts, however their hemodynamic configuration can vary greatly depending upon characteristics of the nidus, i.e., its compactness; resistance of feeding arteries; capacity of draining veins; and the presence of focal weaknesses in the form of aneurysms [2]. Based upon these factors, the lack of capillary resistance in AVMs will result in alterations in blood pressure and shear force across feeding arteries, intranidal vessels, and draining veins thereby promoting venous arterialization and adaptive remodeling, and may be asymptomatic for extended periods of time until rupture occurs [3].

Recent clinical and hemodynamic studies support the concept that the architectural arrangement of venous outflow is related to hemorrhage risk in AVM patients [4]. For example, for AVMs characterized by a short arteriovenous transit distance and high degree of arteriovenous coupling, it has been observed that the pressure and shear forces developed during arteriolarization of venous walls can be transmitted back to the draining veins. It appears that transient limitation of outflow from draining veins, particularly if those veins are located superficially and non-redundantly, can provide a retrograde signal to the nidus resulting in increased pressure sensitivity and thus an increased risk of hemorrhage even in relatively small AVMs [5]. An additional risk factor is the presence of aneurysms in association with AVMs. These aneurysms are focal structural vulnerabilities in AVMs that are embedded in compact arteriovenous shunts. The locations of aneurysms associated with AVMs are typically near curvatures or branches of the intranidal vasculature and are subjected to localized hemodynamic stresses. Rupture of these aneurysms has been associated with hemorrhagic presentations and rupture liability [6].

AVMs occurring in the posterior convexity region of the cerebral hemisphere are of particular interest because of the typical location of hemorrhages in this area results in a characteristic and predictable pattern of neurological deficit. This includes contralateral visual field deficits and impairments in visuospatial attentional functions and may also include pyramidal tract deficits secondary to subcortical pathway compression by hematoma or mass effect created by peri-lesional edema [7]. From a neurosurgeon’s perspective, AVMs located in the posterior convexity region of the cerebral hemisphere may be amenable to a direct microsurgical approach. This is possible given the typically superficial location of the pial arterial supply and the parasagittal location of the draining veins. A direct microsurgical approach to AVMs can be achieved by careful sequential planning of inflow control, nidus devascularization, and vein-last disconnection. Additionally, aneurysm-associated structural vulnerabilities should be identified and managed during surgery [8].

The purpose of this report is to detail a step-wise approach to localize the site of bleeding in a ruptured AVM patient using a combination of bedside neurological examination and catheter-based angiographic imaging. Furthermore, we will detail a three-dimensional reconstruction of the nidus with associated aneurysms and single superficial parasagittal drainage. While our case study does not provide sufficient evidence to draw causal relationships between the described features of the AVM and its propensity to rupture, we believe that description of the AVMs angioarchitecture and microstructural features will facilitate a rupture directed operative strategy to achieve complete cure of the AVM while preserving function of the posterior cortex.

## 2. Case Presentation

This 22-year-old male had no prior cerebrovascular events and no history of substance use which might have affected his nervous system. The individual was fully functional as evidenced by a premorbid modified Rankin Scale (mRS) of 0 and symptoms developed quickly, over several minutes and these included a “thunderclap” headache that reached maximal intensity in less than 10 min, three episodes of projectile vomiting, severe photophobia and an acute decrease in level of alertness that continued to progress over the next few hours. Upon admission to our facility, the patient was somnolent but responsive to verbal stimuli with a GCS of 11 (E3V3M5), brainstem reflexes were intact (normal pupillary response, normal corneal reflex, cough reflex intact, no evidence of decorticate/decerebrate posture), and the FOUR score was 12/16 (Eyes = 3, Motor = 4, Brainstem = 4, Respiration = 1). The patient’s state of alertness worsened over the initial four hours and this prompted us to perform frequent neurologic assessments while obtaining emergent imaging of the brain.

Upon completing a thorough neurologic exam, we determined that the patient was experiencing a right hemisphere lateralizing posterior convexity syndrome. Strength testing of the left upper and lower extremities revealed a pyramidal pattern (Medical Research Council (MRC) = 4/5 for the left upper limb and MRC = 3/5 for the left lower limb). Early development of hypertonia (increased muscle tone), hyperreflexia (increased tendon reflexes) and a positive Babinski response on the left were also observed. The NIH Stroke Scale (NIHSS) was 18 and this was due primarily to contralateral motor weakness (left arm = 3, left leg = 3) and posterior cortical dysfunction (visual field deficit = 2) along with additional points attributed to reduced levels of consciousness. Using the confrontation method to test visual fields, the patient demonstrated a left homonymous hemianopia with macular sparing, and when able to focus attention, a neglect probe showed 40% left-sided cancelation asymmetry. Automated pupillometry showed preserved light reactivity with minor asymmetry (NPi-R = 4.1; NPi-L = 3.8; anisocoria = 0.3 mm), indicating that the patient’s pupil reactivity supported hemispheric pressure physiology without evidence of failure of the brainstem.

Laboratory tests revealed no abnormalities in coagulation or platelet count (INR = 1.0; platelets = 245 × 10^3^/μL), and only mildly elevated inflammatory marker values (WBC = 14.2 × 10^3^/μL; 85% neutrophils; CRP = 18 mg/L) and no evidence of metabolic disturbance. Due to the patient’s age, sudden onset, and lateralizing posterior convexity syndrome deficits, we proceeded with an emergency hemorrhage-first imaging approach. Non-contrast CT demonstrated a large right posterior lobar intraparenchymal hemorrhage (ICH) and we immediately proceeded to obtain neurovascular imaging to determine if an underlying structural lesion existed and to guide definitive management.

**Figure 1:**
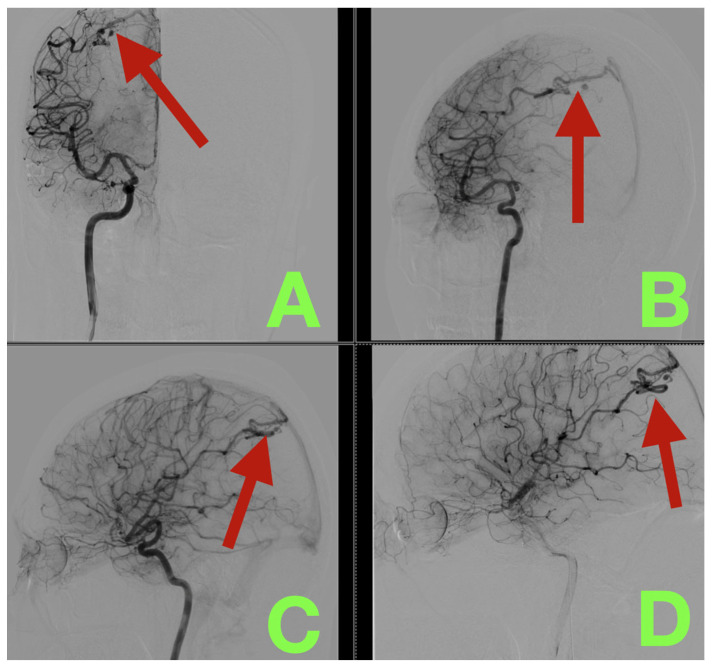
Preoperative digital subtraction angiography of a ruptured right occipital pial arteriovenous malformation. (**A**): AP projection after right ICA injection shows a compact right occipital convexity nidus (arrow) supplied by distal right MCA cortical branches, with early arterial-phase venous filling. (**B**): Lateral projection confirms posterior convexity localization and rapid arteriovenous transit (arrow). (**C**): Oblique view delineates the feeder–nidus interface (arrow) and compact shunt architecture. (**D**): Late venous phase highlights a single superficial cortical draining vein (arrow) emptying parasagittally into the SSS, with no deep venous drainage component.

**Figure 2:**
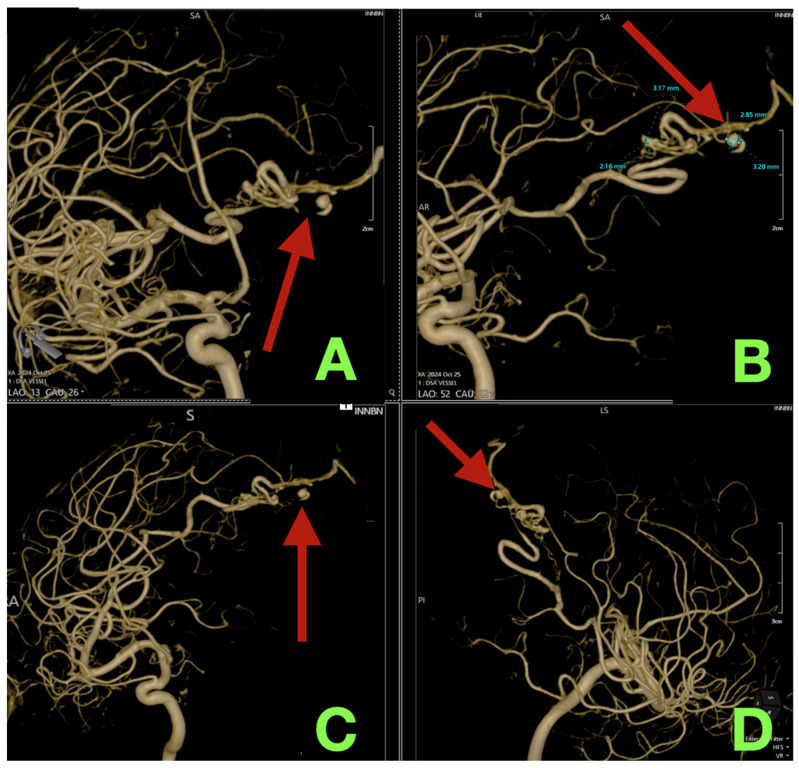
Three-dimensional rotational angiography reconstructions defining nidus micro-architecture and intranidal rupture weak points. (**A**): Oblique 3D overview confirms a compact right occipital nidus measuring approximately 8 × 3 mm (arrow), supplied by right MCA pial afferents. (**B**): Magnified 3D close-up identifies two intranidal aneurysms (annotated) measuring approximately 3 × 2 mm and 3 × 3 mm (arrow), embedded within the shunt axis and representing likely rupture substrates. (**C**): Alternate 3D projection clarifies continuity of dominant MCA cortical feeders into the nidus (arrow). (**D**): Lateral-oblique reconstruction reaffirms exclusive superficial venous drainage via a single cortical vein emptying into the SSS (arrow), defining the solitary outflow corridor that constrained operative sequencing.

Right internal carotid artery digital subtraction angiography (DSA) with multiple views demonstrated a solitary right occipital pial AVM with a compact, clearly defined nidus measuring approximately 8 × 3 mm. The AVM was supplied solely by distal cortical branches of the right middle cerebral artery (MCA). Early venous filling was evident and high flow through the arteriovenous connection was observed. Venous drainage was exclusively superficial through a solitary parasagittal cortical draining vein that drained directly into the superior sagittal sinus (SSS) and there was no deep venous drainage (Figure 1). Three dimensional (3-D) reconstructions created from contrast-enhanced images confirmed the micro-architecture of the nidus and identified two saccular aneurysms within the nidus, each measuring approximately 3 × 2 mm and 3 × 3 mm, respectively. Again, we confirmed that there was solitary superficial drainage to the SSS (Figure 2). These features were felt to be clinically important as they would constrain the order of operation (preservation of the draining vein until complete inflow control was established) and both aneurysms likely served as rupture substrates.

Due to the acute deterioration secondary to the hemorrhage and the superficial nature of the lesion along with solitary superficial drainage, a single stage microsurgical strategy was selected: hematoma-corridor decompression followed by complete excision of the AVM with feeder-first, vein-last disconnection, while maintaining the integrity of parasagittal venous structures and all posterior cortex and posterior pathways. Intraoperative physiological targets were maintained (SBP = 110–130 mmHg; PaCO2 = 35 mmHg; temperature = 36.0 °C; Hb ≥ 10.0 g/dL; Glucose = 80–120 mg/dL). A tailored 6 × 8 cm parieto-occipital craniotomy was performed, reaching to within 5 mm of the SSS without entering it. The two parasagittal bridging veins that drained the superior parietal region were dissected and preserved. The dura was tense and well perfused and was incised in a pedunculated semi-circular manner with the base directed towards the midline. Cortical entry utilized the intergyral sulcus cleft between the superior and inferior parietal lobules to minimize transgression of posterior association cortex and to enter the rupture corridor. The hematoma was located beneath the cortex, approximately 15 mm from the surface and evacuation produced moderate cerebrospinal fluid (CSF) egress through the occipital horn. Evacuation of approximately 50 mL of clot restored cortical pulsatility and produced good brain relaxation (score of 1 on a 1–4 scale).

Following evacuation, circumferential dissection identified a compact subpial nidus with a distinct border. We identified four major arterial feeders that entered the nidus from distal right MCA branches, primarily from the angular and temporo-occipital regions and entered at the sulcal lip. Two transit arteries that appeared to represent en-passage architecture were preserved after confirming that they did not originate from the nidus. The three other pedicles were skeletonized and clipped flush to the nidus. No temporary clipping was required. Once inflow was controlled, the nidus became increasingly darker and softer. The two intranidal aneurysms (3 × 2 mm and 3 × 3 mm) were then treated with low power bipolar shrinkage after reducing inflow. A fibrinous clot cap consistent with the rupture site was observed. The sole draining vein was preserved until we were able to confirm cessation of shunt flow utilizing indocyanine green angiography; after a 4 min observation period confirmed stable de-arterialization and absence of residual pulsatility, the vein was divided. Hemostasis was achieved using bipolar coagulation and layered hemostatic matrix while avoiding thermal injury to the posterior cortex and parasagittal venous structures. Closure was watertight, the bone flap was returned to its original position, and an epidural drain was placed.

The patient was cared for in the Neuro-ICU post-operatively with elevation of the bed (30°), strict normocapnia (PaCO2 = 36 mmHg/EtCO2 = 34 mmHg) and tight blood pressure control (SBP = 105–120 mmHg; MAP = 75–82 mmHg). The use of analgesics and anti-emetics were employed to prevent increases in blood pressure secondary to pain or nausea (paracetamol IV; ondansetron). Seizure prophylaxis was provided (levetiracetam 1000 mg/day for 8 weeks). A short course of steroids was administered (dexamethasone 4 mg q6h tapering over 5 days). The patient underwent frequent neurologic evaluations (every 2 h) early post-operatively. The epidural drain was removed on Post Operative Day #2 (POD#2) without evidence of bleeding (output = 80 mL/24 hr; no collection).

The patient demonstrated a rapid improvement in his neurological status. The patient regained stable wakefulness and was extubated 90 min after anesthesia with a GCS of 14 (E4V4M6). On POD#1, the patient’s FOUR score was 16/16 (E4M4B4R4). The patient’s motor function improved such that strength in the left upper and lower limbs were MRC 5/5 and 4/5, respectively, by POD#3; the patient’s pronator drift resolved and his tone normalized. The patient’s visual/attentional deficits improved as evidenced by the fact that the left homonymous defect diminished to a residual left superior quadrantanopia by POD#2 and the patient’s neglect burden decreased from 40% preoperatively to 5% on star cancelation by POD#4. The patient’s pupillometry remained symmetrical (NPi-R = 4.2; NPi-L = 4.2; anisocoria = 0.1 mm). No seizures or recurrent hemorrhage occurred.

**Figure 3:**
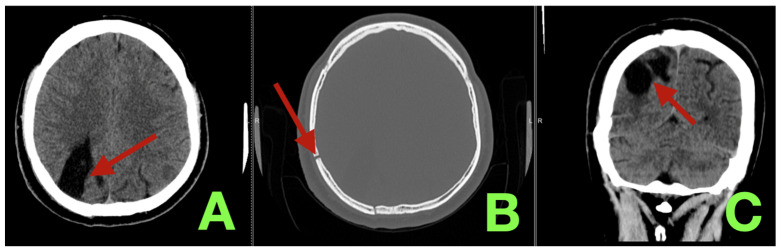
Immediate postoperative non-contrast CT. (**A**): Axial parenchymal window showing a sharply delimited right parieto-occipital postoperative cavity (arrow) replacing the evacuated hematoma and excised AVM nidus, containing expected low-density CSF/pneumocephalus admixture with a thin rim of reactive peri-cavitary hypodensity and no hyperdense re-bleed or new mass effect. (**B**): Axial bone window confirming anatomically stable right parieto-occipital craniotomy margins (arrow) with a re-seated flap and no early epidural or subdural collection. (**C**): Coronal reconstruction demonstrating the posterior convexity resection bed (arrow) with a small air–fluid level typical of immediate postoperative pneumocephalus, preserved hemispheric symmetry, and no evidence of acute hydrocephalus or renewed midline shift.

Immediate post-operative non-contrast CT scan demonstrated a sharply demarcated right parieto-occipital evacuation-resection cavity with expected early CSF/pneumoccephalus admixture and only a thin rim of edema without rebleeding, hydrocephalus, epidural collection or increased mass effect (Figure 3). Laboratory trends were unremarkable: reactive peaks were observed on POD#1 (WBC = 16.1 × 10^3^/uL; neutrophils = 82%; CRP = 32 mg/L) and normalized by the time of discharge (WBC = 8.5 × 10^3^/uL; CRP = 5 mg/L on POD#7). Hemoglobin remained stable (13.8 -> 11.2 g/dL) and electrolyte values were maintained (Na = 138–142 mmol/L; K = 3.9–4.2 mmol/L). The patient was mobilized from assisted sitting on POD#1 to independent ambulation on POD#5; the patient received rehabilitative care focused on visuospatial occupational therapy (four sessions).

**Figure 4:**
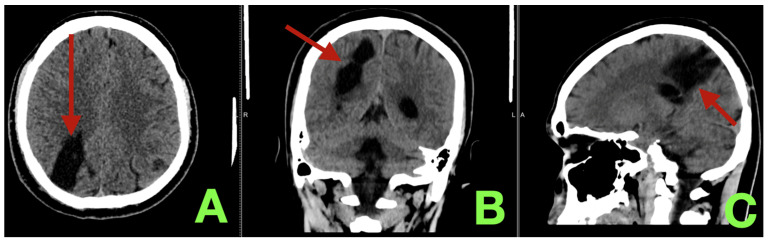
Three-month follow-up non-contrast CT. (**A**): Axial parenchymal window demonstrating a well-circumscribed right parieto-occipital encephalomalacic-gliotic cavity (arrow) corresponding to the healed hematoma–nidus corridor, with smooth margins, no residual hyperdensity, and no recurrent mass effect. (**B**): Coronal reconstruction confirming posterior convexity confinement of the resection bed (arrow), symmetric ventricular configuration, and absence of delayed hydrocephalus or periventricular injury. (**C**): Sagittal reconstruction showing superficial location of the mature resection cavity (arrow) without progressive edema, cystic expansion, or new hemorrhagic change, consistent with a stable, complication-free postoperative evolution and durable AVM excision.

The patient was discharged on POD#7 with stable neurological status (NIHSS = 2; mild left leg drift; mRS = 1; Barthel index = 95/100). The patient was cautioned against strenuous physical activity/Valsalva maneuvers for six weeks and to gradually resume full time university studies over four weeks. On CT imaging at 3 months, there was no evidence of delayed edema, new infarct, extra-axial collection, ventricular enlargement or re-hemorrhage (Figure 4).

Functionally, the patient resumed full time university studies with an mRS of 0 and a Barthel index of 100/100. Perimetric testing confirmed a persistent left superior quadrantanopia; neglect testing normalized (0% asymmetry) and cognitive screening scores remained high (MoCA = 29/30). This case is presented to highlight how intranidal weak points and non-redundant superficial drainage may concentrate hemodynamic stress even in a small posterior convexity AVM and can meaningfully influence presentation and operative sequencing. While conclusions are necessarily limited by the single-case design, careful correlation between hemorrhage topography, angioarchitecture, and microsurgical strategy may assist clinicians in selecting timely curative approaches that prioritize functional preservation.

## 3. Discussion

The ruptured posterior convexity pial AVM in the young adult falls into a clinical concern area; i.e., a nidus can have an anatomically “small” size but be a “high energy” from a hemodynamic standpoint and the rupture characteristics may depend on micro-architectural stress concentrators and/or outflow constraints as opposed to the size of the nidus itself. The patient had a compact right occipital AVM (nidus size = 8 × 3 mm), with two aneurysms within the nidus and one superficial parasagittal draining vein to the superior sagittal sinus resulting in a rupture grade intracranial hypertension cascade with immediate hyperacute arousal failure and sharply localized parietal–occipital cortical/subcortical dysfunction. Original contemporary studies in the area of hemodynamics support the apparent “size–severity paradox”: hemorrhage presentation is associated with adverse intranidal flow states and venous mechanics including local pressure extremes, high shunt fraction, and absence of redundant drainage, as opposed to simply being dependent upon the size of the nidus [9,10]. Intranidal quantitative angiographic frameworks which include behavior of the nidus (“lesion filling”, or “clearance”) demonstrate that delayed intranidal wash-out and micro-stasis are predictive of hemorrhage presentation and thus suggest that rupture risk is due to behavior at the micro-compartment level rather than size of the nidus at the macro-level [11]. Below we outline recent original research in the area of rupture biology/management rationale for small posterior convexity AVM’s (Table 1).

The first structural feature contributing to the likelihood of the initial rupture in this case was the dual intranidal aneurysm complex. Repeatedly identified in earlier studies as the predominant sites of rupture for AVMs (especially those embedded in the nidus), aneurysms create “pressure cooker” environments where pressure and shear gradient forces are maximized, and there is minimal reserve for adaptive remodeling [21]. When located in compact AVMs, intranidal aneurysms are extreme examples of localized curvature and branching in an impedance poor arteriovenous network; transitory inflow surges are less likely to be dissipated across a large plexus and thus more likely to concentrate into these weak points [22]. Thus, two 3 mm intranidal aneurysms within an 8 mm nidus likely created a dual “failure landscape” for this AVM, providing a site where short cycle surges from distal MCA pedicles were likely to be translated into wall stress on an aneurysmal dome. The intraoperative discovery of thin walled domes with an adherent clot cap at a weak point locus provide anatomical face validity to this interpretation, but it is an architectural inference rather than a probabilistic statement [23].

A second structural feature contributing to the likelihood of the initial rupture was the presence of a solitary superficial draining vein. Numerous studies have demonstrated that single drainage AVMs are not simply simplified networks; they are outflow constrained systems in which changes in transient venous impedance will result in the transmission of pressure spikes directly into the nidus. Both computational and morpho-hemodynamic studies of draining veins have provided additional evidence supporting this concept, demonstrating that rupture presenting lesions exhibit more adverse flow profiles in terms of wall shear heterogeneity and acceleration through length limited or caliber limited segments of the draining vein [14]. Even without visual obstruction on static angiography, dependence upon a single superficial parasagittal draining vein likely resulted in increased intranidal pressure at the moment a previously stable intranidal aneurysm destabilized, thereby explaining the sudden intracranial hypertension collapse in a young low compliance cranial vault [24].

This case represents the intersection of current debates regarding optimal timing and strategy for treatment of ruptured AVMs. Recently published comparative cohort studies examining the safety and efficacy of early definitive microsurgical resection versus delayed treatment in patients with ruptured low grade AVMs have shown that early resection can be accomplished safely when a hematoma corridor exists and the angioarchitecture is surgically amenable, and that functional outcomes are equivalent to those seen with delayed treatments, while eliminating the risks of interval re-rupture present with all latency dependent treatments [25]. Utilizing the hematoma cavity as a biologically honest dissection plane, our single stage strategy utilized the rupture cavity to facilitate reversal of compliance collapse and decrease nidal wall stress initially via evacuation of the hematoma, followed by feeder first devascularization with a last step being division of the draining vein. This sequence of events has been demonstrated throughout important microsurgical series to mirror operative physiology and to provide safe curative results, especially in superficial Spetzler-Ponce A architectures without deep drainage [26].

Hematoma removal has its own physiologic impact that can be included when explaining how rapidly this patient improved after surgery. For rupture of posterior convexity AVMs; there is a significant factor contributing to neurologic decline from the large lobar hematoma by way of space-occupying lesion (mass effect), tissue displacement in the immediate area of the hemorrhage, and decrease in intracranial compliance (particularly in younger individuals whose compensatory ability to accommodate volume increases within their cranial vault is decreased) [27]. The early decompression of the patient’s hematoma most probably relieved the pressure on the brainstem and allowed for anatomic restoration of cortically and sub-cortically located areas that had been functionally impaired due to surrounding edema rather than anatomically destroyed. Therefore, the improvements noted post-operatively reflect the effective removal of the arteriovenous malformation causing the shunting, as well as the removal of the primary pressure source contributing to the patient’s decline, thereby demonstrating the two fold benefit of a hematoma-corridor approach to therapy: establishment of normal physiologic conditions of the intracranial space and creation of an anatomic plane free from complications which will allow for the complete removal of the AVM [28].

Surgical literature emphasizes that the intraoperative risk is not uniformly distributed even among low grade AVMs. Studies of predictors of significant blood loss and intraoperative instability in contemporary microsurgical series demonstrate that high flow cortical feeders, fragile subpial nidal planes, and aneurysmal weak points are all strongly predictive of intraoperative hemorrhage, and that utilizing bipolar dominant hemostatic techniques during circumferential devascularization can minimize the amount of intraoperative bleeding [20]. Although the estimated blood loss in our case was reasonable, we intentionally planned our operative choreography around these principles: early decompression to minimize stress on weak points, division of feeders flush with the nidus at sulcal entry zones to protect en-passage arteries, ongoing physiological monitoring of venous devascularization, and a mandatory observational period prior to division of the draining vein. These maneuvers likely contributed to the uncomplicated intraoperative course and lack of complications in this rupture setting [29].

Finally, in addition to the angioarchitectural features described above, there is an increasing number of original studies examining the immune and biomarker signature of AVMs prone to hemorrhage. It appears that reactive leukocytosis and CRP elevation are common in rupture presenting AVM patients, representing an acute neuro-inflammatory response to hemorrhage and mass effect, and not a causative systemic diathesis. Modern studies examining the vascular wall and omics based studies describe endothelial activation programs, macrophage rich inflammatory remodeling and molecular signatures of unstable angiogenic plasticity in the vicinity of rupture presenting AVMs [30]. While these findings currently do not dictate the use of biomarkers in decision making at the bedside, they do represent a near future direction in which microarchitectural risk factors and molecular phenotype may be used for risk stratification and/or adjunctive therapies. Until that day arrives, in low grade compact AVMs with intranidal aneurysms and a single draining vein, logical consideration of weak points remains the most actionable rupture predictor, and biomarkers should be considered contextual information [31].

Treatment of ruptured AVMs continues to be multimodal, yet the majority of recent original outcome studies demonstrate that microsurgical resection provides the highest immediate cure rate for low grade convexity lesions, especially after hemorrhage when a conduit corridor exists. Radiosurgery can provide long term obliteration, but does so with a latency dependent risk of hemorrhage that is unacceptable in a young rupture phenotype with visually identifiable weak points. Embolization of AVMs is critical in the treatment of high grade, eloquent, deep, and/or residual lesions, and can also be used to reduce flow in selected cases prior to definitive surgical resection [32]. However, in very compact cortical nidi with intranidal aneurysms, partial embolization may not sufficiently neutralize the rupture substrate and may actually introduce new impedance gradients within the nidus. Given the distal MCA cortical afferents and the single superficial drainage in our patient, a surgically direct topology existed allowing for definitive resection of both the nidus and the aneurysmal weak points in a single setting, thereby obviating the need for latency dependent risk windows [33].

Endovascular embolization may provide a useful additional approach to manage selected cases of ruptured AVMs; it is especially beneficial in order to occlude specific weak points (i.e., flow related or intranidal aneurysms), or to reduce the amount of shunt inflow into the AVM prior to surgery, thus providing a safer environment for surgeons during their dissection of the AVM [34]. However, the feasibility of using this type of procedure to treat posterior convexity AVMs will depend upon the location and diameter of the distal cortical feeding pedicles, as well as the surgeon’s ability to effectively penetrate the nidus without damaging the vessels that are within the nidus. If the nidus is located in a compact area of the distal brain and is fed by a small number of terminal branches of the MCA, then partial embolization may result in some degree of residual shunting, while also affecting the impedance within the nidus and venous outflow from the nidus, which may be important if there is only one pathway for blood to exit the nidus [35]. This patient had a large hematoma that was compressing his brain and required immediate decompression, which, combined with his surgically favorable anatomy and need to remove all of the aneurysmal weak points located within the nidus of the AVM, led us to recommend a single-stage microsurgical cure for this patient. Therefore, there will be some patients who will benefit from either an endovascular-first or endovascular-adjunct approach depending on the location of the rupture substrate, accessibility of the pedicles, and overall clinical status [36].

As always, several limitations should be clearly articulated. This is a single case report, therefore mechanical inferences made about intranidal aneurysm dominance and one vein pressure sensitivity remain at the level of hypothesis even though supported by modern hemodynamic studies. We did not perform patient specific quantitative flow modeling or lesion filling indices; prospective integration of these types of parameters may increase the accuracy of risk prediction in future posteroconvexity AVMs. Longer surveillance periods are necessary to definitively rule out late recurrence, although the clinical and radiographic follow-up supports durable cure.

In conclusion, this case describes a micro-architecturally “high-risk dense” posteroconvexity AVM (compact occipital nidus, dual intranidal aneurysms, and solitary superficial parasagittal drainage) capable of producing a hemorrhagic syndrome of extreme rapidity in a young adult. The proximity between bedside assessment and angiographic findings allowed for a rupture first, corridor guided microsurgical strategy to restore compliance immediately and eradicate weak points definitively. I hope that this case study provides a fine grained, anatomy and hemodynamics centered model for identifying and treating small posteroconvexity AVMs in whom the rupture biology is determined less by size and more by where stress accumulates within the nidus and where venous outflow is constrained.

## 4. Conclusions

We present this case to expand on a long-standing clinical conundrum: how to diagnose and treat those hemorrhage prone posterior convexity pial brain AVMs where rupture risk does not correlate with nidus size at time of first diagnosis. The combination of clinical presentation, location of bleeding, and angiographic appearance of this patients’ AVM suggest that rupture behavior could be influenced by both nidus characteristics, such as the presence of localized weakness within the nidus and redundancy of venous drainage from the nidus, but also micro-architecture of the surrounding brain.

Although advances have been made in imaging technology, the management of ruptured AVMs in the posterior convexity region has remained relatively unchanged. This case illustrates a general management approach for managing ruptured AVMs in the posterior convexity region; namely, rapid anatomically based decisions regarding management, early physiological stabilization through the hematoma corridor, and staged microsurgical removal under continuous physiological and vascular monitoring. Due to the limitations of a single case study, the good outcome reported here is consistent with the hypothesis that using a disciplined approach to bedside localization of AVMs, high resolution angiographic characterization of AVMs, and rupture-directed microsurgical dissection can lead to timely and definitive treatment while preserving function of the posterior cortex.

## Figures and Tables

**Table 1:** Compiles contemporary cohorts and technical studies relevant to our case, detailing study design, populations, therapies, outcomes, and the specific practice points that map onto our patient’s compact occipital nidus, dual intranidal aneurysms, and solitary superficial drainage. The table is intended to provide a concise, high-yield evidence scaffold for the discussion, highlighting where current data converge on the mechanistic and surgical principles applied here.

References	Design/Cohort	Key Population	Therapy	Outcomes	Practice-Relevant Notes
[12]	Retrospective surgical cohort	Adults with ruptured and unruptured bAVMs managed microsurgically (large single-center series)	Microsurgical resection (graded by SM/Ponce)	High cure rates for low-grade AVMs; functional outcome driven by grade, rupture status, venous anatomy	Confirms microsurgery as definitive modality for low-grade convexity AVMs, especially when a rupture corridor exists; supports your single-stage cure logic.
[13]	Comparative original study (early vs. delayed)	Ruptured bAVMs with ICH, stratified by timing	Early definitive surgery via hematoma corridor vs. delayed elective resection	Early resection not inferior in functional outcome when low-grade and surgically accessible; avoids rebleed window	Directly reinforces your corridor-first strategy: decompression converts pressure emergency into safe anatomical dissection without waiting for “cool-down.”
[14]	Multicenter adult cohort	AVMs with single vs. multiple draining veins	Multimodal management tracked by angioarchitecture	Single-drainage AVMs show higher hemorrhagic presentation and worse venous hemodynamics	Matches your case constraint: a solitary SSS vein is a pressure amplifier, not a benign simplification; mandates vein-last choreography.
[15]	Original CFD + angiographic hemodynamic study	Ruptured vs. unruptured AVMs with quantified venous flow parameters	Hemodynamic modeling of draining veins	Rupture associated with adverse venous profiles (higher shear heterogeneity/flow acceleration)	Supports the concept that rupture risk is venous-microphysics-linked, explaining catastrophic collapse despite small nidus size.
[16]	Prospective quantitative DSA registry	Supratentorial AVMs assessed with QDSA; focus on venous outflow lesions	Imaging-based risk stratification (no intervention assigned)	Venous aneurysms/outflow pathology correlate with hemorrhage and unstable transit patterns	Backs the idea that micro-compartment outflow behavior dominates rupture risk over nidus diameter; helpful when framing your dual-aneurysm/single-vein “high-risk-dense” topology.
[17]	Original cohort on AVM-associated aneurysms	AVMs with feeder/intranidal aneurysms (recent high-resolution series)	Natural history + treated subsets	Intranidal aneurysms strongly associate with hemorrhagic presentation and act as dominant weak points	Mechanistic foundation for your case’s dual intranidal aneurysms being the rupture substrate even in an 8 × 3 mm nidus.
[18]	Single-center original surgical outcomes study	Consecutive intracranial AVM resections, majority ruptured	Microsurgical management with modern intraop verification	Favorable long-term mRS for low-grade ruptured AVMs; low permanent morbidity when venous preservation respected	Emphasizes the same controllable variables you executed: feeder-first devascularization, en-passage preservation, delayed venous division.
[19]	Original morphologic predictor cohort	Adult bAVMs stratified by arterial afferent count + venous drainage	Imaging-risk association	Higher afferent complexity and adverse venous patterns independently associate with rupture	Gives a modern quantitative lens to describe why even compact AVMs can harbor rupture-grade energy, complementing your bedside-to-angiography logic.
[20]	Original predictive microsurgical series	Low-grade bAVMs resected after hemorrhage	Microsurgical resection; predictors of outcome	Favorable mRS mainly determined by grade, compactness, superficial drainage, and corridor availability	Cleanly aligns with your risk-benefit framing for immediate cure in a superficial posterior nidus.

## Data Availability

The data presented in this study are available on request from the corresponding author. The data are not publicly available due to privacy and ethical restrictions related to patient confidentiality.
